# Unveiling the metabolomic profile of growth hormone deficiency children using NMR spectroscopy

**DOI:** 10.1007/s11306-024-02217-9

**Published:** 2025-02-07

**Authors:** Eftychia A. Aggelaki, Aristeidis Giannakopoulos, Panagiota D. Georgiopoulou, Styliani A. Chasapi, Alexandra Efthymiadou, Dimitra Kritikou, Dionisios Chrysis, Georgios A. Spyroulias

**Affiliations:** 1 Department of Pharmacy, School of Health Sciences, University of Patras, 26504 Rio, Greece; 2 Division of Endocrinology Department of Pediatrics, Medical School, University of Patras, 26504 Rio, Greece

**Keywords:** Growth hormone deficiency, Metabolomics, NMR spectroscopy, IGF-1

## Abstract

**Introduction:**

The diagnosis of Growth Hormone Deficiency (GHD) during childhood has been the subject of much controversy over the last few years. Aiming to accurate medical treatment, there is a need for biomarker discovery.

**Objective:**

To characterize the metabolic profile of GHD children, examine the effect of GH administration on the metabolic signature, and investigate the correlations between metabolites and IGF-1.

**Methods:**

Nuclear Magnetic Resonance (NMR)-based untargeted and targeted metabolomic approach applied to study the metabolic profiles of children with GHD. Plasma, serum, and urine samples were collected from twenty-two children diagnosed with GHD and forty-eight age matched controls from the Pediatric Endocrinology Unit of the University Hospital of Patras. Experimental data were examined by both multivariate and univariate statistical analysis.

**Results:**

The results of this pilot study revealed a different metabolic fingerprint of children with GHD in comparison to age-matched healthy individuals. However, the detected alterations in the metabolite patterns before and after GH treatment were subtle and of minor discriminative statistical power.

**Conclusions:**

This study provides evidence that metabolome plays a pivotal role in GHD, but large-scale multicenter studies are warranted to validate the results.

**Supplementary Information:**

The online version contains supplementary material available at 10.1007/s11306-024-02217-9.

## Introduction

Growth Hormone Deficiency (GHD) is a disorder caused by inadequate synthesis and/or secretion of growth hormone (GH) from the anterior pituitary gland (Badaru & Wilson, 2004). GHD, approximately, affects one in every 4.000 to 10.000 children and includes a group of disorders with various etiologies (Dattani & Malhotra, 2019). Complete GHD may be the result of surgical removal of pituitary gland in the case of a tumor (e.g., craniopharyngioma) or GH gene deletion. Many other causes, affecting the GH gene or other pituitary transcription factors involved in GH secretion may result in partial deficiency, usually defined by the results of GH provocation clinical tests. The most prevalent clinical sign of GHD in children is growth failure, which is translated to short stature or/and slowed growth in height and its therapy involves subcutaneous injections of recombinant human GH (Grimberg et al., 2017).

The pattern-release of GH is complex and is characterized by secretory bursts, with two thirds of the total daily GH secretion produced at the first episode of slow-wave sleep. Growth Hormone releasing Hormone (GHRH) and somatostatin are the most important hypothalamic peptide hormones, while several factors might alter the regulation of GH, including stress, exercise and nutritional status (Caputo et al., 2021). GH exerts pleiotropic effects on multiple organs such as muscle, bone and heart and diverse metabolic pathways, either directly or indirectly. GH binds to GHR of cells and stimulates the liver and other tissues to produce insulin-like growth factors (IGFs), especially IGF-1 (Aguiar-Oliveira & Bartke, 2019). The binding of IGF-1 to its receptor, IGF-1R, is followed by activation of a tyrosine-kinase-mediated intracellular signaling pathway, which leads to increased metabolism, anabolism, cellular replication and growth.

Metabolomics, considered one of the most rapidly developing fields of omic-sciences in recent years, is a powerful approach for in-depth understanding of the biological mechanisms of diseases, and may enhance our diagnostic and prognostic potential (Bruzzone et al., 2023; Chasapi et al., 2022; Christopoulou et al., 2023; Georgakopoulou et al., 2020; Georgiopoulou et al., 2022; Katsila et al., 2021; Matzarapi et al., 2022; Preter & Verbeke, 2013; Vignoli et al., 2022). It involves the characterization and quantification of small molecules, within biological systems, such as cells, tissues, organs or an entire organism and the differentiation of their levels appearing in cases of pathological situations. Due to the variation of the metabolome throughout an individual’s lifespan, which reflects the physiological state, the interpretation of metabolomics data is a challenging task. However, such data remain rich in biological information (Clish, 2015). Mass spectrometry (MS) and Nuclear Magnetic Resonance (NMR) spectroscopy are considered the leading analytical approaches for revealing the metabolic state of an organism (Markley et al., 2017). NMR-based analysis of complex biological samples is a non-destructive technique providing sensitivity, repeatability, and demanding limited sample preparation (Markley et al., 2017).

Recent studies have shown the specific effects of GH on metabolism when GH is over or under-produced. Acromegaly, a disease state with increased secretion of GH, shows a decrease in branched-chain amino acids (BCAAs) which is inversely correlated to serum IGF-1 (Biagetti et al., 2019). BCAAs promote skeletal muscle growth by their GH induced increased uptake and can also be used as substrate in gluconeogenesis (Neinast et al., 2018). The metabolic pathways of glycerophospholipid, sphingolipid and linoleic acid seem also to be affected by increased levels of GH (Wang et al., 2020). On the other hand, studies on GHD patients have shown reduced fatty acids and changes in different amino acids like glutamic acid and cysteine (Höybye et al., 2014).

It is known that GH replacement therapy in GHD patients reduces body fat, increases lean muscle mass, and improves the lipid profile by lowering total cholesterol and low-density lipoprotein (LDL) levels (Yang et al., 2023). However, at the metabolome level, GH therapy induces only modest changes in diverse metabolites, including glutamic acid, cysteine, and oleic acid, with no specific metabolite being strongly correlated to IGF-1 (Höybye et al., 2014).

In this study, we attempt to obtain a detailed view on the metabolic fingerprint of children diagnosed with GHD and describe the differences in its profile 3 months after the initiation of therapy with recombinant GH. Plasma, serum, and urine samples of twenty-two GHD children (n = 22) were analyzed by performing untargeted Nuclear Magnetic resonance (NMR)-based metabolomic analysis and compared with forty-eight age-matched controls (n = 48).

Urine has been demonstrated to be a valuable sample matrix across multiple omics fields, regarding the plethora of extracted biological information and the non-invasive sample's collection. As a biological waste material, urine typically contains low levels of proteins, and higher of metabolites from a variety of small molecule classes, including alkaloids, amino oxides, amino acids, bile acids, biogenic amines, carboxylic acids, cresols, hormones and related compounds, indoles and derivatives, nucleobases and related compounds, vitamins and cofactors, acylcarnitines, cholesteryl esters, and diglycerides. Despite significant advancements in metabolite identification, not all detected features in urine can be confidently annotated. This limitation in reference databases can impede the interpretation of statistical findings. Research has demonstrated that urine composition is not only formed by underlying health conditions or dietary intake, but also by demographic factors. For instance, studies have shown that both gender and age have a statistically significant effect on the urinary metabolome (Thévenot et al., 2015), emphasizing the complex nature of this biological fluid. Plasma and serum are both derived from the liquid component of blood after the removal of cells. However, they differ in composition and preparation. Plasma is collected from blood treated with anticoagulants, while serum is obtained after the blood has clotted, resulting in the absence of clotting factors. Consequently, plasma and serum exhibit slightly distinct metabolic profiles, due to variations in metabolite stability, composition and the biological processes related to blood coagulation. Hence, we aim to analyze both plasma and serum metabolome since it offers complementary insights into an organism’s metabolic status. To our knowledge, this is the first time that the effect of GH administration has been investigated in a subset of patients following untargeted NMR metabolomics analysis using the three biofluids (plasma, serum, and urine).

## Methods

### Ethics statement

This study was approved by the General University Hospital of Patras human research ethics committee and all procedures performed in studies involving human participants were in accordance with the ethical standards of the institutional and/or national research committee and with the 1964 Helsinki declaration and its later amendments or comparable ethical standards.

### Population selection

Seventy children, twenty-two diagnosed with GHD (10 girls and 12 boys) and forty-eight healthy children (30 girls and 18 boys) were enrolled in this study, after obtaining informed consent from the patients and/or their parents. GH diagnosis was validated based on clinical and laboratory grounds according to consensus guidelines form GH Research society (Society, 2000). GHD was defined by stimulated GH levels < 10 ng/ml in 2 consecutive GH provocation tests. Moreover, to assess the effects of GH therapy over time, follow-up samples were collected from 15 out of 22 GHD children, three months after the commencement of GH administration. Comprehensive baseline characteristics of all participants are given in Table 1.

**Table 1:** Baseline characteristics of 70 participants

Characteristics	Healthy controls (n = 48)	GHD group (n = 22)	3 months after initiation of treatment (n = 15)
Male/female	18/30	12/10	9/6
Age (years ± SD)	7.75 ± 2.21	6.94 ± 3.13	7.21 ± 2.88
Weight (kg ± SD)	26.58 ± 13	20.19 ± 8.47	n/a
Height (cm ± SD)	121.031 ± 17.89	109.78 ± 18.72	n/a
BMI (kg/m^2^ ± SD)	17.25 ± 4.12	16.03 ± 1.79	n/a
IGF-1 (SDS ± SD)	n/a	− 0.88 ± 1.42	1.418 ± 1.02

*BMI* body mass index *IGF-1* insulin-like growth factor-1, *SD* standard deviation, *n/a* not available

### Sample collection and preparation

Plasma, serum, and urine samples were collected under fasting conditions, stored and prepared for NMR analysis according to the below-mentioned protocols, with adaptations as described by Bernini et al. (2011). Both sample storage and NMR sample preparation were carried out at the Department of Pharmacy, University of Patras.

From each donor, blood plasma and serum samples were withdrawn using BD Vacutainer® K3-EDTA (K3-ethylenediamine tetra-acetate) spray coated tubes and BD Vacutainer® SSTTM. Serum samples were gently mixed by inverting the tube 5 times. Then they were left at room temperature for 30 min and allowed to clot in an upright position. Blood samples were centrifuged at 1500×g for 10 min at room temperature. For each sample, the supernatant was aliquoted in 3 fractions of 600 μL using 2 mL cryovials and stored at − 80 °C. Urine samples were stored immediately after collection at 40 °C for maximum of 2 h to avoid cell breaking upon ice crystal formation. Urine samples were centrifuged at 2500×g for 5 min at room temperature and the supernatant from each sample was aliquoted in three fractions of 1 mL each and were stored in 2 mL cryovials at − 80 °C.

### NMR sample preparation

Before NMR analysis, frozen aliquots were thawed at room temperature. Plasma and serum samples (300 μL) were mixed with 240 μL of sodium phosphate buffer (0.14 M Na_2_HPO_4_, 0.5 mM 4,4-dimethyl-4-silapentane-1-sulfonic acid (DSS), 4% NaN_3_ in H_2_O, pH 7.4) and 60 μL D_2_O.Urine samples (540 μL) were mixed with 60 μL of potassium phosphate buffer (1.5 M KH_2_PO_4_, 100% v/v D_2_O, 0.05 mM DSS and 4% NaN_3_, pH = 7.4) (Suarez-Diez et al., 2017). After vortexing, 550 μL of each mixture was transferred into a 5 mm NMR tube (Bruker BioSpin srl).

### NMR data acquisition

All spectra were recorded at a Bruker Avance III HD 700 MHz spectrometer equipped with a 5 mm cryogenically cooled 5.0 mm 1H/13C/15N/D Z-gradient probe. To reveal all the detectable ^1^H signals of metabolites for plasma, serum and urine samples, two one-dimensional (1D) ^1^H NMR spectra were acquired using a standard NOESY (NOESYpresat) pulse sequence for water suppression for all the three specimens, while the 1D ^1^H CPMG (Carr-Purcell-Meiboom-Gil) pulse sequence with a presaturation routine only for plasma and serum samples. Also, the homonuclear 2D ^1^H *J*-resolved (*J*-res) correlation NMR experiment with presaturation routine, typical for metabolomics experiments, acquired for each sample. More specifically, plasma and serum NMR spectra were acquired at 37 °C, while urine samples at 25 °C. All the acquisition parameters for the 1D ^1^H NOESY (noesygppr1d; Bruker), the 1D ^1^H CPMG (cpmgpr1d; Bruker), and the 2D ^1^H *J*-res (jresgpprqf; Bruker) experiments were identical to those applied in the study of Matzarapi et al. (2022).

### Data processing

All NMR spectra were manually processed using Topspin software (version 4.1.1, Bruker Biospin srl), transforming them appropriately for the subsequent steps of the metabolomic analysis. Specifically, calibration, phase and baseline corrections were performed (Puchades-Carrasco et al., 2016). Plasma and serum NMR spectra were calibrated on the anomeric proton of a-D-glucose at 5.24 ppm, (d), (considered as an inherent internal standard), while for the urine NMR spectra were aligned on the DSS at 0.00 ppm, (s) (Pearce et al., 2008). The NMR spectral data were converted into a data matrix (bucket table) using Amix 3.9.12 software (Bruker BioSpin) following the “bucketing” method. Blood plasma and serum 1D ^1^H CPMG spectra were segmented into buckets of 0.04 ppm width in the spectral region from 0.7 to 8.85 ppm. ^1^H signals of water (4.68–5.10 ppm) were excluded from the plasma and serum bucket table. Also, the resonances of the anticoagulant ethylenediamine tetra-acetate (EDTA) (2.55–2.62, 2.68–2.71 ppm, 3.07–3.24 ppm, 3.59–3.63 ppm) were excluded from the plasma bucket table.

Urine 1D ^1^H NOESY NMR spectra were segmented into buckets of 0.02 ppm width across the spectral region from 0.7 to 10.5 ppm, to reduce spectral complexity (due to the highly overlapping proton signals). The resonances of DSS (1.73–1.79 ppm, 2.90–2.93 ppm), urea (5.50–6.24 ppm) and water (4.61–5.16 ppm) were excluded from the urine bucket table as well.

### Statistical analysis and computational methods

Metabolite identification was performed utilizing the free version of Chenomx NMR Suite 8.6, the databases Human Metabolome Database (HMDB), Biological Magnetic Resonance Bank (BMRB) and data from the literature (Wishart et al., 2007). Signals were assigned using both 1D and 2D NMR spectra, through examination of all the spectral characteristics such as chemical shifts (*δ*), multiplicity and *J* coupling constants. Multivariate analysis (MVA) was the first step to statistically investigate biological data, discover possible outliers and classification trends. MVA was performed using the online available software MetaboAnalyst 5.0 (Pang et al., 2021). For plasma and serum samples the unsupervised method of Principle Component Analysis (PCA) and the supervised method of Partial Least Squares Discriminant Analysis (PLS-DA) were applied by performing Pareto scaling and normalization by median and range scaling, respectively. For urine samples, to overcome the differences in urine dilution, the application of normalization methods was also required. Probabilistic Quotient Normalization (PQN) and range scaling were chosen as methods for the best statistical preprocessing. The model was validated examining the parameters R^2^ and Q^2^ after a tenfold cross validation. The values of these parameters are characteristic for the model’s fitness and predictability. Univariate statistical analysis was the key to analyze independently the non-overlapping metabolites’ peaks (Saccenti et al., 2014). This targeted analysis was conducted using the programming language R (R studio), via the non-parametric Kruskal-Wallis test. Metabolites with *p*-value < 0.05 after the False Discovery Rate (FDR) correction were characterized as statistically significant. Correlation analysis between statistically significant metabolites and IGF-1 was performed using Pearson Correlation test and the statistical significance was set at *p* < 0.05.

### Pathway analysis

Metabolite Pathway analysis (MetPA) was performed by MetaboAnalyst 5.0, shedding light on the biological mechanisms and biochemical pathways which are involved and altered in GHD. The names of the statistically significant metabolites were imported as input, the hypergeometric test was chosen as enrichment method, the relative betweenness centrality was preferred for topological analysis while the Homo Sapiens was the selected pathway library from KEGG (Ren et al., 2015).

## Results

### Plasma metabolomic analysis

**Fig. 1:**
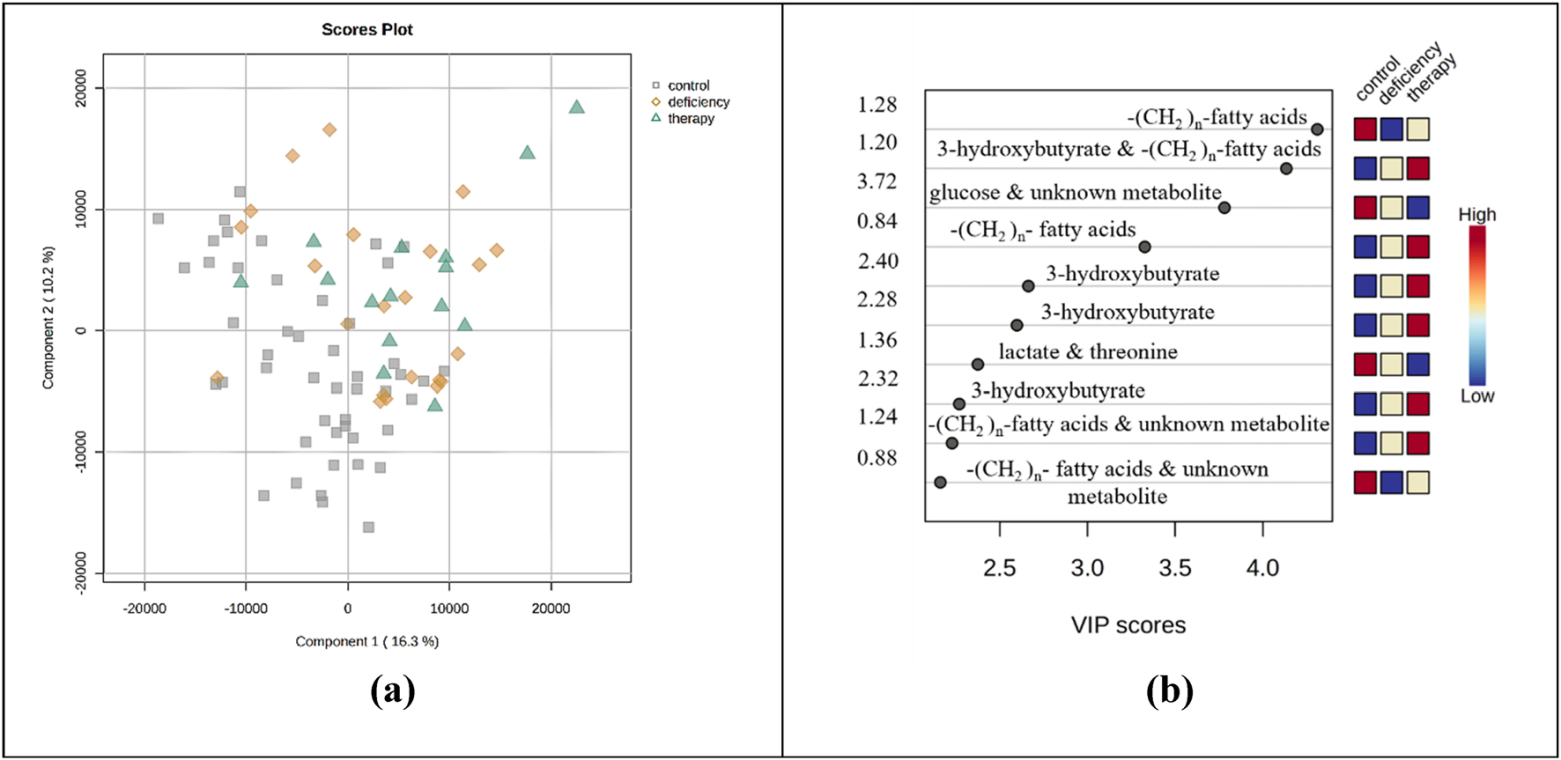
Multivariate analysis of 1D ^1^H CPMG plasma spectra obtained from children diagnosed with GHD before (yellow rhombus) and during the 3 months of GH replacement (green triangles) as compared with healthy controls (grey squares). **a** 2D PLS-DA scores plot indicates that the controls differ from GHD patients regardless of therapy. **b** Rank of the top ten variables identified by the PLS-DA according to the VIP scores on the x-axis. Colored boxes on the right correspond to the relative concetration of each specific variable in each group

**Fig. 2:**
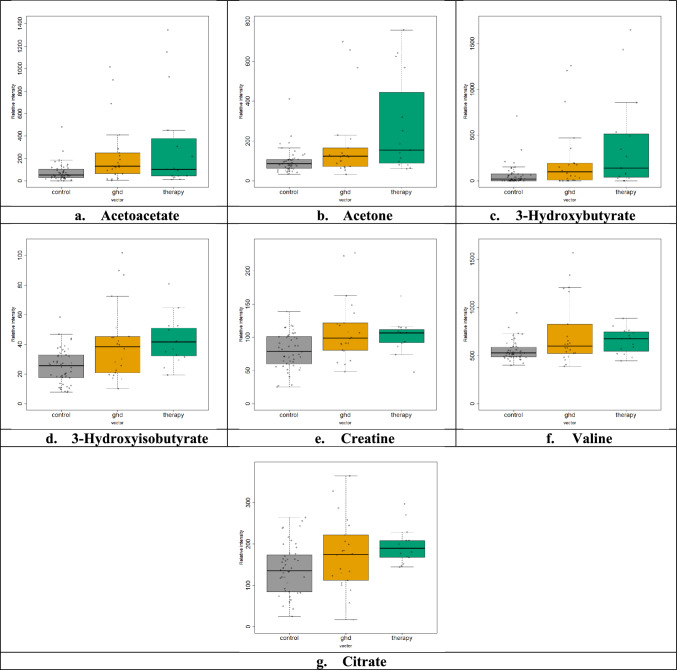
Univariate statistical analysis (**a–g**). Boxplots displaying the variation of relative concetration for each one of the seven plasma metabolites, which significantly differ between the three groups: healthy controls (grey, “control”), GHD children before the initiation of therapy (yellow, “ghd”) and GHD children after the 3 months of GH replacement (green, “therapy”)

For the needs of the current study, a total of eighty-five plasma samples (n = 85) were screened: Forty-eight (n = 48) samples from age-matched healthy controls (30 girls and 18 boys), twenty-two (n = 22) samples from children diagnosed with GHD (10 girls and 12 boys), and fifteen (n = 15) samples from children diagnosed with GHD three months after the initiation of GH therapy (6 girls and 9 boys). A total of 30 metabolites were detected and successfully assigned in plasma NMR spectra from GHD children (Table S1).The CPMG NMR data resulted from the bucketing procedure were applied as input for the PCA (Fig. S1) and PLS-DA method, which provided a reliable model (Fig. 1a). The tenfold cross validation test demonstrated a Q^2^ value of 0.20 and an R^2^ value of 0.40 for the third latent variable. For the classification of plasma metabolic profiles among the three groups, the characteristic *n* methylene groups of fatty acids (1.28, 1.20, 1.24 and 0.88 ppm, –(CH_2_)_n_–), 3-hydroxybutyrate (1.20 ppm, –CH_3_, d and 2.40, 2.28, 2.32 ppm, –CH_2_–, dd), glucose (3.72 ppm, –CH–, m) and lactate and threonine (1.36 ppm, –CH_3_, d) were found to be statistically significant according to the VIP scores (VIP > 1) (Fig. 1b). Further validation of these results was achieved through univariate statistical analysis. Boxplots from the univariate analysis, representing the differentiation of the relative intensity among the three groups corroborated the results of MVA (Fig. 2). Specifically, the boxplots highlighted that the levels of the following seven metabolites, acetoacetate (2.28 ppm, –CH_3,_ s), acetone (2.24 ppm, –CH_3_, s), 3-hydroxybutyrate (2.31 ppm, –CH_2_–, dd), 3-hydroxyisobutyrate (1.07 ppm, –CH_3,_ d), creatine (3.92 ppm, –CH_2_–, s) valine (1.04 ppm, –CH_3_, d) and citrate (2.65 ppm, –CH_2_–, d) are increased in GHD children compared to healthy controls. These metabolites have been identified as statistically significant with *p*-value < 0.05 after FDR correction (Table 2). Moreover, minor alterations were observed between the metabolic profiles of untreated and treated children over the three months of GH treatment.

**Table 2:** Results of the univariate statistical analysis from plasma metabolites

Metabolites	3 groups	Control-GHD	Control-therapy	Before-after therapy
	*p-value*			
Acetoacetate	0.011	0.010	0.122	0.781
Acetone	0.005	0.030	0.007	0.252
3-Hydroxybutyrate	0.005	0.028	0.008	0.385
3-Hydroxyisobutyrate	0.001	0.011	7.0 × 10^–5^	0.45
Creatine	0.005	0.009	0.009	1
Valine	0.018	0.066	0.030	0.804
Citrate	0.005	0.11	0.002	0.216

Seven statistically significant metabolites according to their *p*-values after the comparison between plasma samples obtained from healthy controls and GHD children before and during 3 months of GH replacement

### Serum metabolomic analysis

**Fig. 3:**
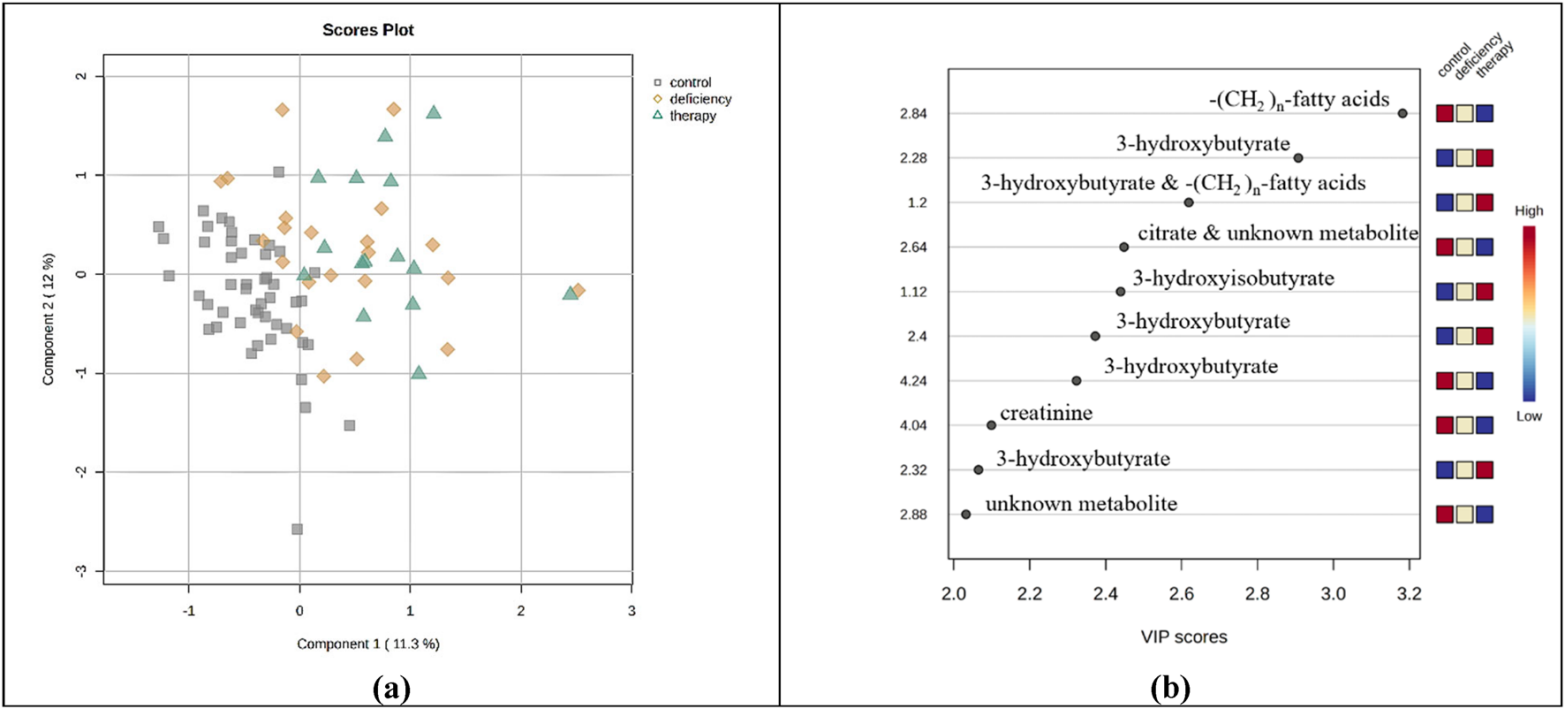
Multivariate analysis of 1D ^1^H CPMG serum spectra obtained from children diagnosed with GHD before (yellow rhombus) and during 3 months of GH replacement (green triangles) as compared with healthy controls (grey squares). **a** 2D PLS-DA scores plot indicates that the controls differ from GHD patients regardless of therapy **b** Rank of the top ten variables identified by the PLS-DA according to the VIP scores on the x-axis. Colored boxes on the right correspond to the relative concetration of the specific metabolite in each group

**Fig. 4:**
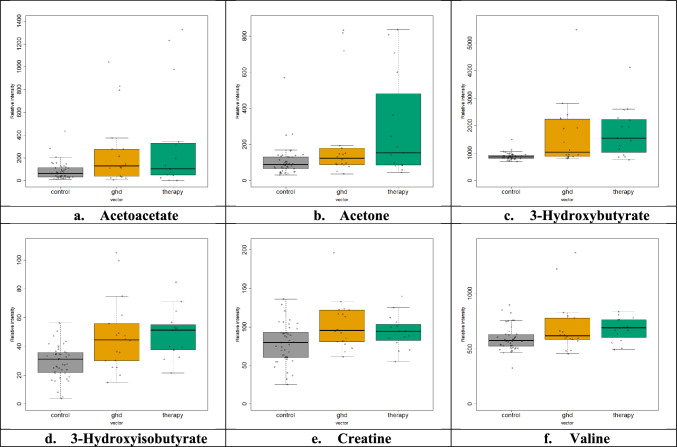
Univariate statistical analysis (**a–f**). Boxplots displaying the variation of relative concetration for each one of the six serum metabolites, which significantly differ between the three groups: healthy controls (grey, “control”), GHD children before the initiation of therapy (yellow, “ghd”) and GHD children after the 3 months of GH replacement (green, “therapy”)

In total, eighty-five serum samples (n = 85) were screened: Forty-eight (n = 48) samples from age-matched healthy controls (30 girls and 18 boys), twenty-two (n = 22) samples from children diagnosed with GHD (10 girls and 12 boys), and fifteen (n = 15) samples from children diagnosed with GHD three months after the initiation of GH therapy (6 girls and 9 boys). A total of 30 metabolites were successfully detected and assigned in serum NMR spectra from GHD children (Table S2). CPMG NMR data were explored via PCA (Fig. S2) and PLS-DA (Fig. 3a), which evaluated with an R^2^ of 0.59 and Q^2^ of 0.41 for the third latent variable, after a tenfold cross validation test. The variables of importance for the PLS-DA classification of serum metabolic profiles indicate the ^1^H peaks which correspond to the bucket 2.84 of the characteristic *n* methylene groups of fatty acids (–(CH_2_)_n_–), 3-hydroxybutyrate (1.20 ppm, –CH_3_, d, 2.28 and 2.40 ppm, –CH_2_–, dd, and 4.24 ppm, –CH–, m), 3-hydroxyisobutyrate (1.12 ppm, –CH_3,_ d), citrate (2.64 ppm, –CH_2_–, d) and creatinine (4.04 ppm, –CH_2_–, s) (Fig. 3b). Univariate analysis, in accordance with the results of the multivariate statistical approach, revealed higher levels of acetoacetate, acetone, 3 -hydroxybutyrate, 3-hydroxyisobutyrate, creatine and valine in GHD children (Fig. 4, Table 3), presenting a pattern of group differentiation similar to plasma ^1^H NMR metabolic profile.

**Table 3:** Results of univariate statistical analysis of serum metabolites

Metabolites	3 groups	Control-GHD	Control-therapy	Before-after therapy
	*p*-value			
Acetoacetate	0.048	0.05	0.12	0.86
Acetone	0.017	0.03	0.02	0.53
3-Hydroxybutyrate	1.0 × 10^–4^	0.002	3.0 × 10^–4^	0.41
3-Hydroxyisobutyrate	4.0 × 10^–4^	0.006	6.0 × 10^–4^	0.41
Creatine	0.019	0.017	0.07	0.56
Valine	0.041	0.11	0.04	0.37

Six statistically significant metabolites according to their *p*-values after the comparison between serum samples obtained from healthy controls and GHD children before and during 3 months of GH replacement

### Urine metabolomic analysis

**Fig. 5:**
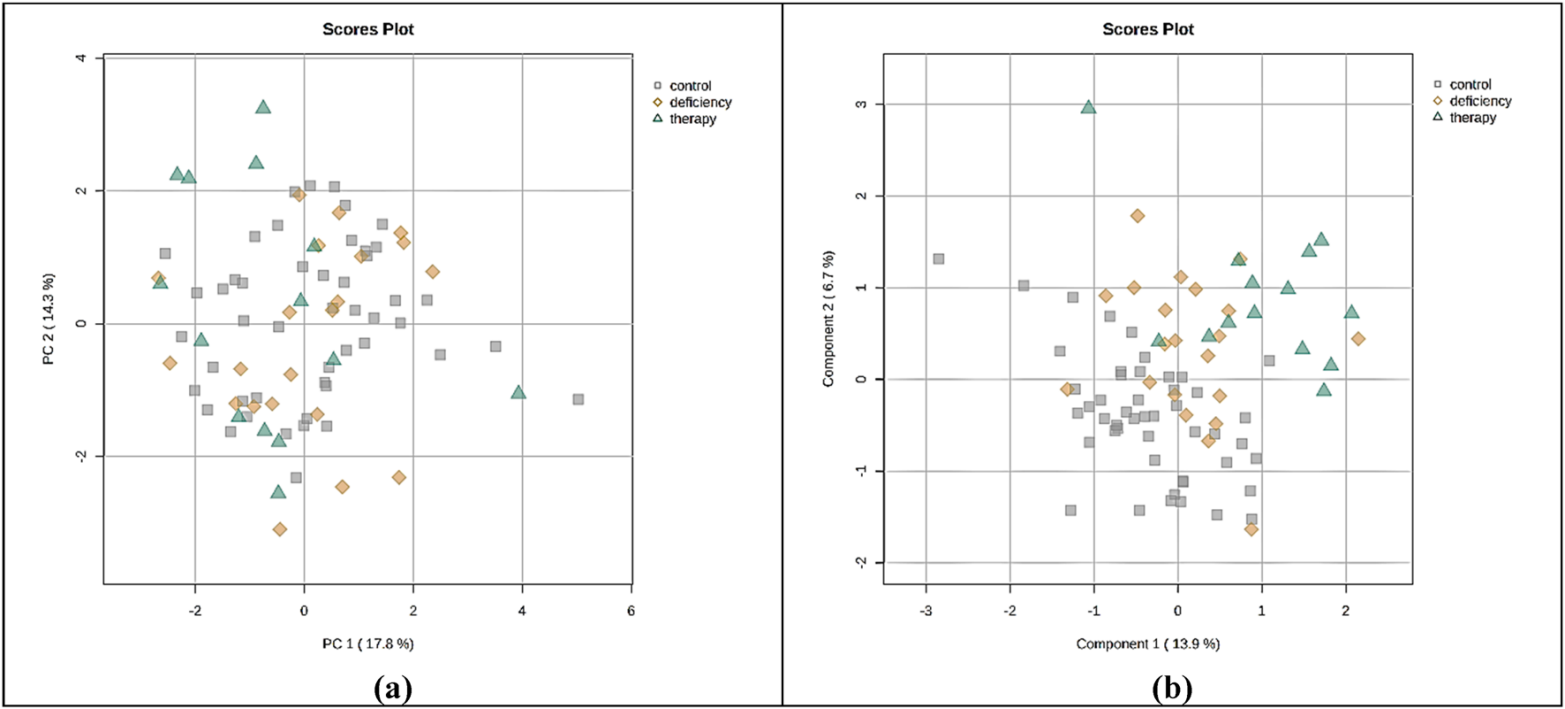
Multivariate analysis of 1D ^1^H NOESY urine spectra obtained from children diagnosed with GHD before (yellow rhombus) and during 3 months of GH replacement (green triangles) as compared with healthy controls (grey squares). **a** 2D PCA scores plot with the first and second principal component **b** 2D PLS-DA scores plot indicate that the controls differ from GHD patients regardless of therapy

A total of 32 metabolites were successfully detected and assigned in the urine NMR spectra from GHD children (Table S3). Initial investigation of the urinary metabolic profiles performed via PCA analysis imprinted the increased heterogeneity of ^1^H NMR spectra, leading to a low percentage (37.9% at the third principal component) of the model’s explained cumulative variance (Fig. 5a). Moreover, the PLS-DA model revealed low discrimination efficacy in classification of the three groups, with the tenfold cross validation test resulting in an R^2^ value of 0.73 and Q^2^ value of 0.24 for the third latent variable (Fig. 5b). The high dimensionality, which characterizes the urine metabolomic datasets, relative to the number of samples, often leads to overfitting models in statistical analysis. Due to the complex nature of urine as a biological fluid-varying in pH, metabolites, proteins, hormones, ions and other small molecular weight molecules and its highly variable composition both between individuals and within the same individual over time, only the metabolites with ^1^H NMR discriminant signals were examined for statistical significance. Univariate statistical analysis of 17 metabolites (which did not present any overlapping features in ^1^H NMR spectra) was performed. The relative concentrations of the metabolites were compared, yet none of them demonstrated statistical significance. To investigate alterations in metabolite levels a fold change analysis was conducted, revealing the relative differences between healthy controls and GHD children (comparison A). Additionally, comparisons between GHD children before and during 3 months of GH treatment (comparison B) as well as GHD children during 3 months of GH treatment and healthy controls (comparison C) were performed and are highlighted in Table 4.

**Table 4:**
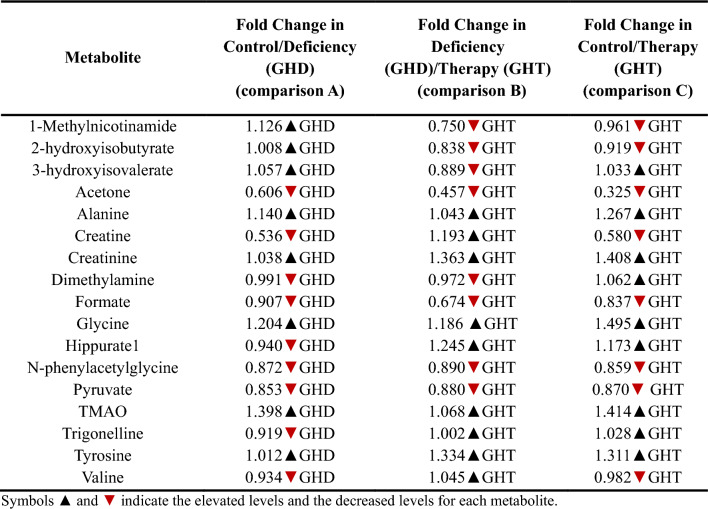
Fold change analysis of the 17 urine metabolic features detected among healthy controls and GHD children before and during 3 months of GH treatment

### Correlation analysis between potential biomarkers and IGF-1

**Fig. 6:**
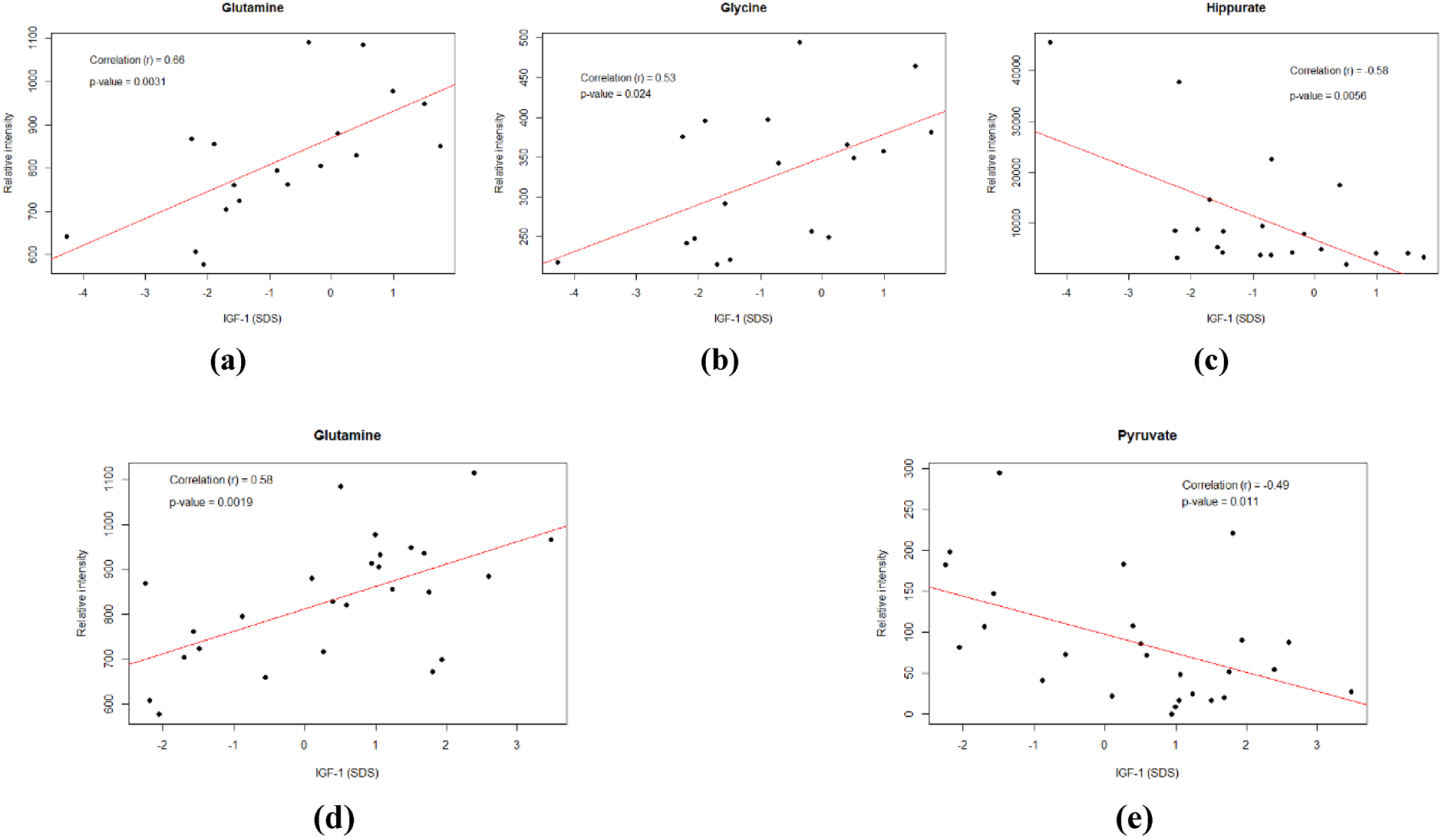
2D Scatter plots (**a–e**) with fit lines between different metabolites and IGF-1 (SDS). r: Spearman’s rank correlation coefficient and the corresponding *p*-value is shown in each plot. **a–c** Plots refer to serum glutamine, serum glycine and urine hippurate in correlation to IGF-1 (SDS) in GHD group while **d, e** plot shows glutamine and pyruvate changes in GHD group 3 months after initiation of therapy with GH in correlation to IGF-1

To investigate any correlation between the observed metabolites’ changes and the levels of IGF-1, Pearson’s correlation analysis was conducted using the values of IGF-1 and the semiquantitative data of univariate analysis about the levels of the examined metabolites for each sample group, GHD children and GHD children 3 months after the initiation of treatment. The results are displayed in Fig. 6. Before GH therapy in children with GHD, serum levels of glutamine (r = 0.66, *p*-value = 0.0031) and glycine (r = 0.53, *p*-value = 0.024) were significant and positive correlated with IGF-1 (SDS), while urine levels of hippurate (r = − 0.58, *p*-value = 0.0056) were significantly negatively correlated with IGF-1. After 3 months of GH treatment, only serum glutamine (r = 0.58, *p*-value = 0.0019) and serum pyruvate (r = − 0.49, *p*-value = 0.011) showed statistically significant correlations with IGF-1 (SDS).

### Metabolic disturbances induced by growth hormone deficiency

**Fig. 7:**
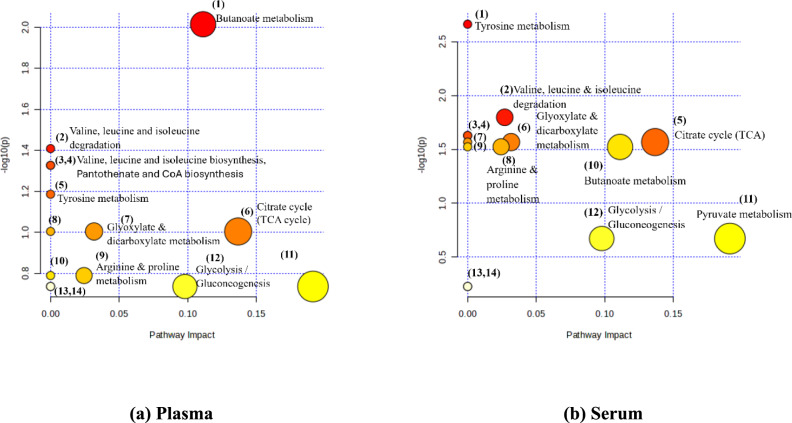
Metabolic pathway analysis map of the GHD NMR plasma (**a**) and serum (**b**) metabolomic data

Pathway analysis was the final approach on metabolomics data, aiming at the correlation of the up-regulated and down-regulated metabolites and the visualization of all the possibly affected metabolic processes. Semiquantitative data of all statistically significant plasma and serum metabolites were implemented into MetaboAnalyst pathway analysis. Plasma metabolome and serum metabolome view are displayed in Fig. 7a, b. Each circle corresponds to an altered metabolic pathway. Color intensity, from white to red reflects the increasing statistical significance, expressed via *p*-value (y-axis), while circle’s diameter represents the pathway impact (x-axis). Furthermore, Tables S4 and S5 show the detailed results about *p*-value with FDR correction and the impact values calculated from pathway topology analysis of plasma and serum metabolome, respectively.

## Discussion

The aim of this study was to determine plasma, serum and urine metabolic profile of children diagnosed by GHD and investigate any effects of 3-months GH therapy on the metabolic signature through NMR spectroscopy. We further examined whether modifications on the levels of certain metabolites are correlated with changes in IGF-1 (SDS). Our findings revealed a distinct metabolic fingerprint in GHD children compared to healthy controls, but the alterations in the metabolome three months after initiation of GH therapy were minor, with no discriminative statistical power.

More specifically, metabolites’ composition was similar either in plasma or in serum. Moreover, the results indicated increased levels of the following three ketone bodies acetoacetate, 3-hydroxyisobutyrate and 3-hydroxybutyrate. These findings suggest an upregulation of ketogenesis, this metabolic pathway that provides an alternative energy source when glucose is scarce. Ketogenesis is typically activated by counterregulatory hormones such as glucagon, growth hormone, and cortisol, which promote fatty acid mobilization for energy production (Kerner & Hoppel, 2004). In the context of GHD the absence of this key regulator may lead to impaired glucose utilization and increased reliance on ketone body production to meet energy demands.

The detailed analysis of the blood metabolome showed that children with GHD had higher circulating levels of valine compared to the control group. Interestingly, previous studies have confirmed lower levels of that metabolite in adults with acromegaly, a condition characterized by excessive GH secretion (Biagetti et al., 2019). This contrast supports the idea of a negative association between GH and the levels of valine.

Moreover, we observed higher levels of creatine among children with GHD, suggesting a potential increase in skeletal mass protein turnover. This finding indicates that GHD may affect muscle metabolism, likely due to alterations in muscle protein synthesis and degradation.

In contrast, plasma and serum glutamine levels were significantly reduced in children with GHD compared to healthy controls. This was supported also by univariate analysis, in where glutamine was assessed independently. GH therapy tended to normalize glutamine levels by increasing them, and, interestingly, these changes were statistically correlated with variations in IGF-1 SDS levels observed before and after initiation of treatment. Decreased glutamine levels have previously been found in a group of children with short stature, which included both GHD and idiopathic short stature cases (Xu et al., 2019).

Furthermore, the NMR metabolomic analysis revealed that many of the metabolites altered in GHD could be classified as fatty acids (FAs). However, the methodology employed in this study focused to the aqueous metabolite content of the biological fluids analyzed. Moreover, this ^1^H NMR analysis provides limited structural information about the presented lipids, which cannot lead to the specific lipid characterization. Recently, Yang et al. (2023), published a detailed metabolic analysis focused on the lipid profile of adult GHD. Their identified signature markers include diacylglycerol (DG), cytidine diphosphate DG (CDP-DG), phosphatidylcholine (PC), phosphatidylethanolamine (PE), phosphatidylinositol, phosphatidylserines, lysophosphatidylcholine, and lysophosphatidylethanolamine (LysoPE), all of which are implicated in pathways such as unsaturated fatty acids biosynthesis, sphingolipid metabolism, glycerophospholipid metabolism, fatty acid elongation, degradation and biosynthesis. Thus, based on our results we could only highlight a reduction in FAs in children with GHD, while GH treatment tented to normalize FAs levels, in accordance with the well-established lipolytic effect of GH (Molitch et al., 2011).

Additionally, we identified all the other pathways involving metabolites with statistically significant levels. More specifically, for both plasma and serum metabolome the affected metabolic pathways included alanine, aspartate and glutamate metabolism, arginine and proline metabolism, butanoate metabolism, citrate cycle, cysteine and methionine metabolism, glyoxylate and dicarboxylate metabolism, glycine, serine and threonine metabolism, the pathway of glycolysis/gluconeogenesis, pantothenate and CoA biosynthesis, pyruvate metabolism, tyrosine metabolism as well as biosynthesis and degradation of valine, leucine and isoleucine.

To evaluate the metabolomic signatures associated with different pediatric health conditions, we aimed to assess the similarities, differences and unique patterns within their respective NMR metabolic fingerprints. Consequently, we compared the results of the present study with the findings of the metabolomic analysis of children with premature adrenarche (PA), a previous research which was conducted using the same analytical approach and methodology (Matzarapi et al., 2022). More specifically, in PA children, plasma levels of alanine, glucose, glycerol, lactate, and leucine, as well as serum levels of glucose, glycerol, glycine, myo-inositol, and serine, were notably different. In contrast, the metabolomic signature associated with growth hormone deficiency (GHD) exhibited a high degree of similarity between plasma and serum samples, characterized by elevated concentrations of acetoacetate, acetone, 3-hydroxybutyrate, 3-hydroxyisobutyrate, creatine, and valine. Regarding the urinary metabolome, the GHD profile revealed differences in a panel of 17 metabolites, while the PA urinary metabolome highlighted four key metabolites: 3-methyl-histidine, hippurate, urocanic acid, and mannitol. Among these, only hippurate appears to be a common marker, showing reduced levels in both PA and GHD children compared to healthy individuals. Based on our observations, nuclear magnetic resonance (NMR) analysis of urinary metabolic profiles, even though it is complicated, has demonstrated enhanced sensitivity when applied to the classification of pediatric metabolic disorders through an untargeted analytical approach.

As a constraint of this study regarding the interpretation of data could be considered the number of participants, which was relatively limited, particularly those who received GH treatment. A more extensive sample size could potentially improve the discrimination between pre and post GH treatment metabolomic profiles; however, the advantage of the paired samples empowers the statistical analysis concerning the differences between the levels of diverse metabolites across all fluids. Given that GH initiates its metabolic actions early, it is expected that the 3-month interval may be adequate to reveal changes. On the other hand, longitudinal data collected at longer intervals may reveal more significant or even different effects.

Overall, it is undeniable that the analysis of the metabolome is quite complex and there have been limited studies to date regarding the GH deficiency, particularly in children. This study establishes a pivotal starting point, providing valuable insights into the relationship between the metabolic profile of children diagnosed with growth hormone deficiency (GHD) and those experiencing typical physiological growth. Additionally, it represents the first metabolome analysis aiming at correlating the impact of growth hormone (GH) therapy on children with GHD pre and post initiation of treatment. The identified IGF-1 associated metabolites can be investigated in future metabolomic analysis as potential biomarkers to monitor the metabolic effect of GH treatment. To reveal all the unique features of the metabolome and its role in the diagnosis and therapy of GHD more studies are required. Future studies should strive for increasing the sample size and examining the effects of GH replacement after six (6), twelve (12) and twenty-four (24) months (with a long term follow up outcomes up to 5 years or so). Moreover, as studies have demonstrated that GHD affects the pathway of biosynthesis of unsaturated fatty acids in adults, lipidomic analysis in pediatric population is also required to confirm these findings in younger ages and provide additional information about children’s lipidomic profiles.

## Supplementary Information

Below is the link to the electronic supplementary material.

Supplementary file1 (DOCX 556 KB)

## Data Availability

No datasets were generated or analysed during the current study.
