# Supplementary material for: Unveiling the metabolomic profile of growth hormone deficiency children using NMR spectroscopy
Source: Metabolomics. 2025 Feb 7;21(1):25. doi: 10.1007/s11306-024-02217-9 (PMC11805833; doi:10.1007/s11306-024-02217-9)
Supplement: Supplementary file 1 — Supplementary file1 (DOCX 556 KB) [file 11306_2024_2217_MOESM1_ESM.docx]

Unveiling the Metabolomic Profile of Growth Hormone Deficiency Children Using NMR Spectroscopy

Eftychia A. Aggelaki^1^, Aristeidis Giannakopoulos^2^, Panagiota D. Georgiopoulou^1^, Styliani A. Chasapi^1^, Alexandra Efthymiadou^2^, Dimitra Kritikou^2^, Dionisios Chrysis^2*^, Georgios A. Spyroulias^1*^

^1^Department of Pharmacy, School of Health Sciences, University of Patras, Rio, 26504, Greece.

^2^Division of Endocrinology Department of Pediatrics, Medical School, University of Patras, Rio, 26504, Greece

*Correspondence: [G.A.Spyroulias@upatras.gr](mailto:G.A.Spyroulias@upatras.gr) (G.A.S); [dchrysis@upatras.gr](mailto:dchrysis@upatras.gr) (D.C).

Supplementary Material

Table S1: ^1^H NMR Chemical Shifts and multiplicities of identified plasma metabolites in GHD children.

| No | **Metabolites** | **^1^H NMR Chemical Shifts*** |
| --- | --- | --- |
|  | 1-methylhistidine | 7.77 (s) |
|  | 3-methylhistidine | 7.05 (s) |
|  | 2-Hydroxybutyrate | 0.88 (t) |
|  | 3-Hydroxybutyrate | 1.20 (d), 2.31 (dd), 2.39 (q), 4.13 (m) |
|  | 3-Hydroxyisobutyrate | 1.07 (d) |
|  | a_1_-acid glycoproteins | 2.04 (s) |
|  | Acetone | 2.24 (s) |
|  | Acetoacetate | 2.28 (s), 3.43 (s) |
|  | Alanine | 1.48 (d) |
|  | Valine | 0.99 (d), 1.04 (d), 2.26 (m) |
|  | Lactate | 1.32 (d),4.11 (q) |
|  | Glutamine | 2.09 (m), 2.41 (m) |
|  | Glutamate | 2.14 (m), 2.36 (m) |
|  | Glycerol | 3.57 (dd), 3.66 (dd) |
|  | Glycine | 3.56 (s) |
|  | Glucose | 3.25 (dd), 3.41 (t), 3.42 (t), 3.46 (m), 3.49 (t), 3.54 (dd), 3.72 (t), 3.73 (dd), 3.77 (q), 3.82 (m), 3.84 (m), 3.90 (dd), 4.64 (d), 5.24 (d) |
|  | Threonine | 1.32 (d) |
|  | Isoleucine | 0.93 (t), 1.00 (d) |
|  | Citrate | 2.53 (d), 2.65 (d) |
|  | Creatine | 3.04 (s), 3.92 (s) |
|  | Creatinine | 3.05 (s), 4.05 (s) |
|  | Leucine | 0.95 (d),0.96 (d) |
|  | Formate | 8.46 (s) |
|  | Acetate | 1.91 (s) |
|  | Proline | 3.34 (m) |
|  | Pyruvate | 2.37 (s) |
|  | Trimethylamine | 2.87 (s) |
|  | Tyrosine | 6.90 (d), 7.20 (d) |
|  | Phenylalanine | 7.34 (m), 7.37 (m),7.42 (m) |
|  | Choline | 3.21 (s) |

*^1^H NMR signals multiplicities: (s) singlet; (d) doublet; (t) triplet; (dd) doublet of doublets; (m) multiplet; (br) broad

| 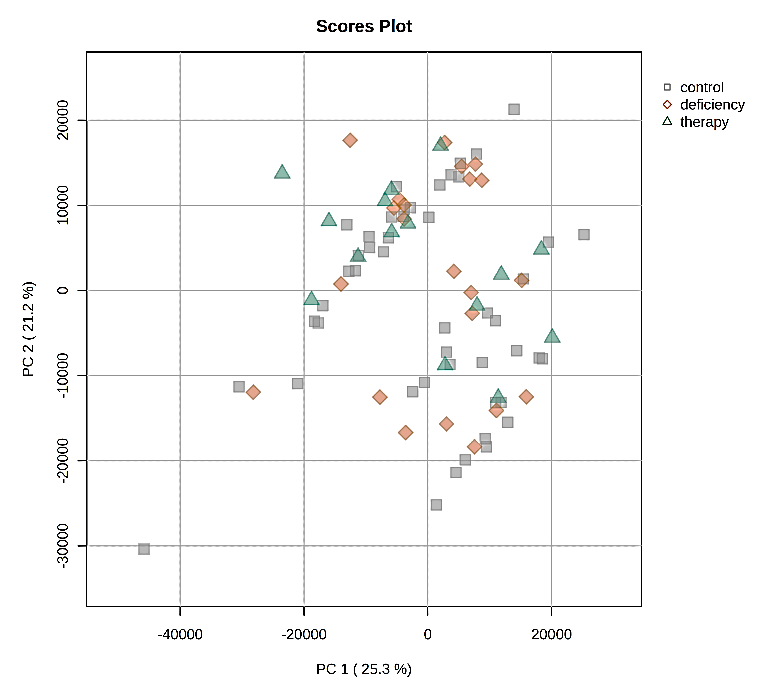 |
| --- |

Figure S1: Unsupervised visualization of cluster tendency. 2D PCA scores plot of 85 ^1^H CPMG NMR plasma spectra obtained from children diagnosed with GHD before (yellow rhombus) and during the 3 months of GH replacement (green triangles) as compared with healthy controls (grey squares).

Table S2: ^1^H NMR Chemical Shifts and multiplicities of identified serum metabolites in GHD children.

| No | **Metabolites** | **^1^H NMR Chemical Shifts*** |
| --- | --- | --- |
|  | 1-methylhistidine | 7.77 (s) |
|  | 3-methylhistidine | 7.05 (s) |
|  | 2-Hydroxybutyrate | 0.88 (t) |
|  | 3-Hydroxybutyrate | 1.20 (d), 2.31 (dd), 2.39 (q), 4.13 (m) |
|  | 3-Hydroxyisobutyrate | 1.07 (d) |
|  | a_1_-acid glycoproteins | 2.04 (s) |
|  | Acetone | 2.24 (s) |
|  | Acetoacetate | 2.28 (s), 3.43 (s) |
|  | Alanine | 1.48 (d) |
|  | Valine | 0.99 (d), 1.04 (d), 2.26 (m) |
|  | Lactate | 1.32 (d),4.11 (q) |
|  | Glutamine | 2.09 (m), 2.41 (m) |
|  | Glutamate | 2.14 (m), 2.36 (m) |
|  | Glycerol | 3.57 (dd), 3.66 (dd) |
|  | Glycine | 3.56 (s) |
|  | Glucose | 3.25 (dd), 3.41 (t), 3.42 (t), 3.46 (m), 3.49 (t), 3.54 (dd), 3.72 (t), 3.73 (dd), 3.77 (q), 3.82 (m), 3.84 (m), 3.90 (dd), 4.64 (d), 5.24 (d) |
|  | Dimethylamine | 2.71 (s) |
|  | Threonine | 1.32 (d) |
|  | Isoleucine | 0.93 (t), 1.00 (d) |
|  | Citrate | 2.53 (d), 2.65 (d) |
|  | Creatine | 3.04 (s), 3.92 (s) |
|  | Creatinine | 3.05 (s), 4.05 (s) |
|  | Leucine | 0.95 (d),0.96 (d) |
|  | Formate | 8.46 (s) |
|  | Acetate | 1.91 (s) |
|  | Proline | 3.34 (m) |
|  | Pyruvate | 2.37 (s) |
|  | Tyrosine | 6.90 (d), 7.20 (d) |
|  | Phenylalanine | 3.12 (dd), 7.34 (m), 7.37 (m),7.42 (m) |
|  | Choline | 3.21 (s) |

*^1^H NMR signals multiplicities: (s) singlet; (d) doublet; (t) triplet; (dd) doublet of doublets; (m) multiplet; (br) broad

| **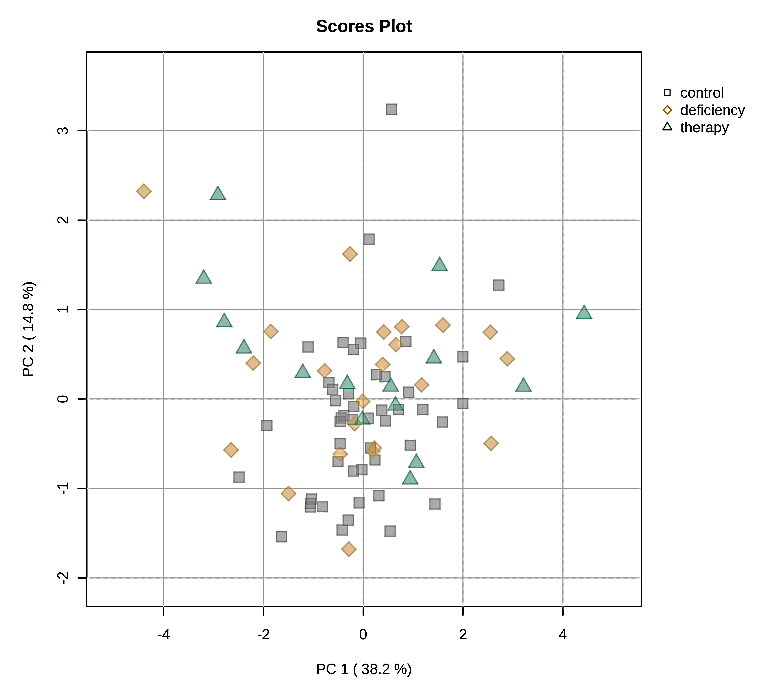** |
| --- |

Figure S2:Unsupervised visualization of cluster tendency. 2D PCA scores plot of 85 ^1^H CPMG NMR serum spectra obtained from children diagnosed with GHD before (yellow rhombus) and during the 3 months of GH replacement (green triangles) as compared with healthy controls (grey squares).

Table S3: ^1^H NMR Chemical Shifts and multiplicities of identified urine metabolites in GHD children.

| No. | **Metabolites** | **^1^H NMR Chemical Shifts*** |
| --- | --- | --- |
|  | 1-methylnicotinamide | 4.46 (s), 8.88 (d), 8.95 (d), 9.26 (s) |
|  | 2-oxoglutarate | 2.99 (t) |
|  | 2-Hydroxyisobutyrate | 1.34 (s) |
|  | 3-Hydroxyisovalerate | 1.25 (s) |
|  | Ethanolamine | 3.14 (t) |
|  | Acetone | 2.22 (s) |
|  | Alanine | 1.46 (d) |
|  | Allantoin | 5.38 (s) |
|  | Valine | 0.97 (d), 1.03 (d) |
|  | Lactate | 1.31 (d) |
|  | Glutamine | 2.12 (m), 2.44 (m) |
|  | Glycine | 3.55 (s) |
|  | Glycolate | 3.93 (s) |
|  | Dimethylamine | 2.71 (s) |
|  | Indoxyl Sulfate | 7.19 (m), 7.26 (m), 7.34 (s), 7.49 (d), 7.69 (d) |
|  | Threonine | 1.31 (d) |
|  | Hippurate | 7.54 (t), 7.62 (t), 7.82 (d) |
|  | Isobutyrate | 1.09 (d) |
|  | Citrate | 2.52 (d), 2.68 (d) |
|  | Creatine | 3.02 (s), 3.91 (s) |
|  | Creatinine | 3.03 (s), 4.04 (s) |
|  | Lactose | 4.44 (d), 5.22 (d) |
|  | Formate | 8.44 (s) |
|  | Ν,Ν Dimethylglycine | 2.93 (s), |
|  | TMAO | 3.25 (s) |
|  | Ν-phenylacetylglycine | 3.66 (s), 7.34 (m), 7.41 (m) |
|  | Acetate | 1.90 (s) |
|  | Urea | 5.76 (s,br) |
|  | Pyruvate | 2.33 (s) |
|  | Taurine | 3.41 (t) |
|  | Trigonelline | 8.07 (m), 8.82 (m), 9.11 (s) |
|  | Tyrosine | 6.88 (d), 7.19 (d) |

*^1^H NMR signals multiplicities: (s) singlet; (d) doublet; (t) triplet; (dd) doublet of doublets; (m) multiplet; (br) broad

Table S4: Detailed results from the plasma pathway analysis of the GHD group’s significant metabolites.

| **No** | **Pathway** | ***p*-value FDR** | **Impact** |
| --- | --- | --- | --- |
| 1. | Butanoate metabolism | 1.35E-01 | 0.11 |
| 2. | Valine, leucine and isoleucine degradation | 1.65E-01 | 0.00 |
| 3. | Valine, leucine and isoleucine biosynthesis | 1.65E-01 | 0.00 |
| 4. | Pantothenate and CoA biosynthesis | 1.65E-01 | 0.00 |
| 5. | Tyrosine metabolism | 1.73E-01 | 0.00 |
| 6. | Citrate cycle (TCA cycle) | 1.73E-01 | 0.14 |
| 7. | Glyoxylate and dicarboxylate metabolism | 1.73E-01 | 0.03 |
| 8. | Alanine, aspartate and glutamate metabolism | 1.73E-01 | 0.00 |
| 9. | Arginine and proline metabolism | 1.84E-01 | 0.02 |
| 10. | Glycine, serine and threonine metabolism | 1.84E-01 | 0.00 |
| 11. | Pyruvate metabolism | 1.84E-01 | 0.19 |
| 12. | Glycolysis / Gluconeogenesis | 1.84E-01 | 0.10 |
| 13. | Cysteine and methionine metabolism | 1.84E-01 | 0.00 |
| 14. | Lipoic acid metabolism | 1.84E-01 | 0.00 |

Table S5: Detailed results from the serum pathway analysis of the GHD group’s significant metabolites.

| **No** | **Pathway** | ***p*-value FDR** | **Impact** |
| --- | --- | --- | --- |
| 1. | Tyrosine metabolism | 3.05E-02 | 0.00 |
| 2. | Valine, leucine and isoleucine degradation | 4.22E-02 | 0.03 |
| 3. | Cysteine and methionine metabolism | 4.22E-02 | 0.00 |
| 4. | Lipoic acid metabolism | 4.22E-02 | 0.00 |
| 5. | Citrate cycle (TCA cycle) | 4.22E-02 | 0.14 |
| 6. | Glyoxylate and dicarboxylate metabolism | 4.22E-02 | 0.03 |
| 7. | Alanine, aspartate and glutamate metabolism | 4.22E-02 | 0.00 |
| 8. | Arginine and proline metabolism | 4.22E-02 | 0.02 |
| 9. | Glycine, serine and threonine metabolism | 4.22E-02 | 0.00 |
| 10. | Butanoate metabolism | 4.22E-02 | 0.11 |
| 11. | Pyruvate metabolism | 2.47E-01 | 0.19 |
| 12. | Glycolysis / Gluconeogenesis | 2.47E-01 | 0.10 |
| 13. | Valine, leucine and isoleucine biosynthesis | 5.92E-01 | 0.00 |
| 14. | Pantothenate and CoA biosynthesis | 5.92E-01 | 0.00 |
